# A novel graphical evaluation of agreement

**DOI:** 10.1186/s12874-022-01532-w

**Published:** 2022-02-20

**Authors:** Jongphil Kim, Ji-Hyun Lee

**Affiliations:** 1 Department of Biostatistics and Bioinformatics, H. Lee Moffitt Cancer Center & Research Institute, Tampa, USA; 2 Department of Oncologic Sciences, University of South Florida, Tampa, USA; 3 Department of Biostatistics, University of Florida, Gainesville, USA; 4 Division of Quantitative Sciences, University of Florida Health Cancer Center, Gainesville, USA

**Keywords:** Agreement, Bland-Altman plot, Concordance correlation coefficient, Graphical evaluation, Limits of agreement, Reference band

## Abstract

**Background:**

The Bland-Altman plot with the limits of agreement has been widely used as an absolute index for assessing test-retest reliability or reproducibility between two measurements. We have observed that in the settings where the relative index such as concordance correlation coefficient (CCC) or intraclass correlation coefficient is employed, the limits of agreement approach may be inconsistent with the scaled index. Particularly, the broad width of the limits of agreement may indicate a lack of agreement when the two measurements are highly concordant but an acceptable difference is not known and the common variance of the data is large. This research aims to create a novel, CCC-based guidance for graphical evaluation of reproducibility or reliability.

**Methods:**

The concordance correlation coefficient is used to create a 100(1-α)% reference band from two measurements. Simulation studies and real examples, including the peak expiratory flow rate data in Bland and Altman’s paper and the test-retest reproducibility data of the Radiomics study, are implemented to assess the use of the reference band.

**Results:**

In the absence of an acceptable difference between measurements, we found that the limits of agreement may not be consistent with the concordance correlation coefficient. Our simulation study results and real data application show that the proposed method can provide practitioners with a novel graphical evaluation that is consistent with results from the concordance correlation coefficient.

**Conclusions:**

Our proposed novel scaled index-based guidance can be used for the graphical evaluation of reproducibility or reliability and may have advantages over the limits of agreement in settings where the concordance correlation coefficient is employed.

**Supplementary Information:**

The online version contains supplementary material available at 10.1186/s12874-022-01532-w.

## Background

In the process of the development of new predictors or features in clinical studies, it is essential to assess how reliable or reproducible they are. The reliability or reproducibility of the features is evaluated by either unscaled summary indices based on absolute difference of measurements, such as the limits of agreement (LoA) [1–3], the coverage probability (CP), the total deviation index (TDI) [4, 5] or scaled summary indices, such as the concordance correlation coefficient (CCC) by Lin [6] or the intraclass correlation coefficient (ICC). If the difference between measurements is interpretable and an acceptable difference has been established (e.g., blood pressure, peak expiratory flow rate in Bland and Altman [1], etc.), unscaled indices should be selected for assessing reliability or reproducibility. However, in cases when the difference is not interpretable or an acceptable difference is not available, the CCC or the ICC have been widely used as scaled indices for two or more continuous measurements. For example, Balagurunathan et al. [7] developed 219 quantitative 3D imaging features derived from computed tomographic (CT) images, which may be useful as prognostic biomarkers in non-small cell lung cancer studies. These imaging features include texture features such as pixel histogram, run length, co-occurrence or 3D-Laws. The difference of these features can be hard to interpret clinically; thus, the acceptable difference for such a feature cannot be predetermined. The CCC was selected to evaluate the reproducibility or reliability of imaging features. More details regarding the definition of repeatability, reproducibility, validity, reliability, and agreement indices for continuous measurements are available in Barnhart et al. [8]. In this paper, we use agreement, reliability, and reproducibility interchangeably since we seek to propose a novel visual tool for assessing agreement between two measurements. The pros and cons of different agreement indices are well compared in Barnhart et al. [9].

The Bland-Altman (B-A) plot with the LoA has been widely used as an absolute index for assessing agreement due to its simplicity and intuitive appeal and it was reported as one of the top 100 most cited papers of all time (Van Noorden et al. [10]). Suppose that *n* pairs of samples (*X*_1*i*_, *X*_2*i*_), *i* = 1, …, *n* are collected independently from a bivariate normal distribution *X* = (*X*_1_, *X*_2_)^*T*^ with mean *μ* = (*μ*_1_, *μ*_2_)^*T*^ and variance-covariance matrix $$\left(\begin{array}{cc}{\sigma}_1^2& \rho {\sigma}_1{\sigma}_2\\ {}\rho {\sigma}_1{\sigma}_2& {\sigma}_2^2\end{array}\right)$$, |*ρ*| < 1. The CCC, *ρ*_*c*_, is expressed as the product of two terms:

*ρ*_*c*_ = *ρC*_*b*,_ $0<{C}_b=\frac{2}{\frac{\sigma_1}{\sigma_2}+\frac{\sigma_2}{\sigma_1}+\frac{{\left({\mu}_1-{\mu}_2\right)}^2}{\sigma_1{\sigma}_2}}\le 1,$where *ρ* is the correlation coefficient and the term *C*_*b*_ measures how far the best-fit line deviates from the perfect concordance line *X*_1_ = *X*_2_. Bland and Altman [1] proposed a residual type plot of the observed pairs of data for evaluation of agreement. The LoA is defined as

$$\overline{d}\pm {t}_{n-1,0.025}{S}_d,$$

where *d*_*i*_ = *X*_2*i*_ − *X*_1*i*_, $\overline{d}=\frac{1}{n}\sum {d}_i$, ${S}_d^2=\frac{1}{n-1}\sum {\left({d}_i-\overline{d}\right)}^2$, *t*_*n* − 1,0.025_ is 100 × (1-0.025) percentile of the t-distribution with *n* − 1 degrees of freedom. The LoA contains nearly 95% of the observed differences, and inference is made by comparing the LoA with the predetermined acceptable difference. The approximate and exact 95% confidence intervals for the LoA were investigated by Bland and Altman [2] and Carkeet [3], respectively. In biomarker studies, including Balagurunathan et al.’s Radiomics study, the reproducibility of the features was frequently evaluated by the CCC, but the B-A plot with the LoA was presented as a graphical illustration of reproducibility. Since the CCC is a scaled or relative index, the LoA in the Bland-Altman plot (an unscaled or absolute index), may not be associated with the CCC values in some settings. Thus, a novel CCC-based guidance for graphical evaluation of agreement will be helpful for the CCC users.

In this paper, we present a CCC-based visual tool for assessing agreement in cases where no acceptable difference is available, and a scaled index is used for evaluating the reliability or reproducibility. We believe that the proposed method provides practitioners with not only guidelines for a descriptive graphical evaluation of agreement, but also with useful information such as recognition of patterns or identification of outliers in the data. The Methods section of this paper shows how a reference band (RB) as a descriptive visual tool is derived from the CCC. The comparisons with the LoA and the association between the proportion of outliers identified in the RB (i.e., % of outliers) and the CCC values are presented in the Results section. A peak expiratory flow rate study from Bland and Altman’s paper and the Radiomics features extracted from 3D CT images in Balagurunathan et al. have been considered as examples to illustrate our approach.

## Methods

Unlike the total deviation index (TDI) and the coverage probability (CP) by Lin [4], Lin et al. [5], and Escaramis et al. [11]; we assume that *C*_*b*_ = 1 (i.e., *μ*_1_ = *μ*_2_ and *σ* = *σ*_1_ = *σ*_2_). Then, $\sqrt{n}\ \left({\overline{X}}_2-{\overline{X}}_1\right)$ is normally distributed with mean 0 and variance 2*σ*^2^(1 − *ρ*), and the estimator *S*_*d*_^2^ for 2*σ*^2^(1 − ρ) is distributed as $2{\sigma}^2\left(1-\uprho \right){\chi}_{\nu}^2/\nu$ with degrees of freedom *ν* = *n* − 1. For a given the correlation coefficient 𝜌, the variable $t=\frac{\sqrt{n}\ \left({\overline{X}}_2-{\overline{X}}_1\right)}{S_d}$ is distributed as a central *t*-distribution with degrees of freedom *ν*. Thus, the probability over a band in $\left({\overline{X}}_1,{\overline{X}}_2\right)$ plane defined as.

$\left\{\left({\overline{X}}_1,{\overline{X}}_2\right),-\infty <{\overline{X}}_1<\infty, -\upomega \le \sqrt{n}\ \left({\overline{X}}_2-{\overline{X}}_1\right)\le \upomega \right\}$, ω = *t*_*ν*, *α*/2_*S*_*d*_,is exactly 1 – 𝛼, assuming *C*_*b*_ = 1. The ω is the half-width of the LoA.

As a graphical tool for assessing agreement, we introduce a 100(1 - 𝛼)% “reference band (RB)” where half-width RB ω_*RB*_ is defined as

$${\upomega}_{RB}={t}_{\nu, \alpha /2}\ {S}_d\sqrt{\frac{1-{\rho}_L}{1-\hat{\rho}}},$$

given confidence level 1 - 𝛼 and degrees of freedom *ν*. Here, ρ_*L*_ is the lower bound of excellent concordance. As $\hat{\upsigma}={S}_d/\sqrt{2\left(1-\hat{\uprho}\right)}$, the half-width of the RB can be written as

$${\upomega}_{RB}={t}_{\nu, \alpha /2}\hat{\upsigma}\sqrt{2\left(1-{\uprho}_L\right)}.$$

**Fig. 1:**
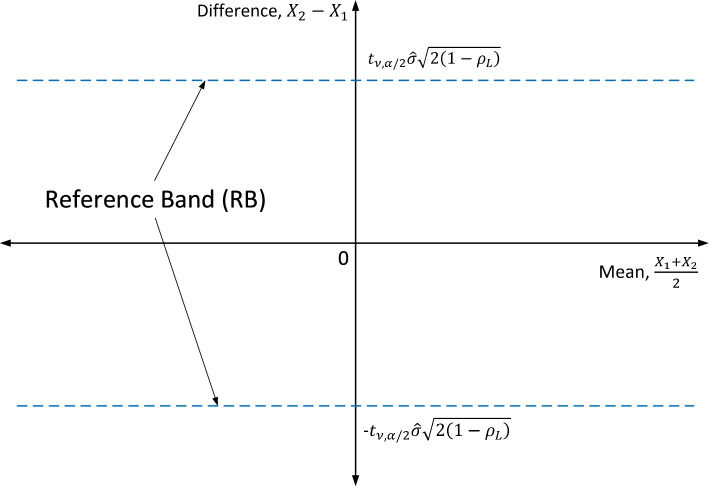
Definition of the reference band (RB) in $\left(\frac{X_1+{X}_2}{2},{X}_2-{X}_1\right)$ plane

Note that the variance of *X*_2*i*_ − *X*_1*i*_ is the same as of $\sqrt{n}\ \left({\overline{X}}_2-{\overline{X}}_1\right)$ and that $\hat{\rho}>{\rho}_L$ iff *ω*_*RB*_ > *ω*, $\hat{\rho}<{\rho}_L$ iff *ω*_*RB*_ < *ω*, and $\hat{\rho}={\rho}_L$ iff *ω*_*RB*_ = *ω*. In other words, if the half-width of the RB is narrower than that of the LoA, then the CCC value will be lower than ρ_*L*_ since ρ_*C*_ ≤ ρ < *ρ*_*L*_. The two lines, *X*_2_ − *X*_1_ =  ± *ω*_*RB*_ in $\left(\frac{X_1+{X}_2}{2},{X}_2-{X}_1\right)$ plane, are the boundary lines of the RB, as illustrated in Fig. 1. This difference vs. average plot would allow us to better investigate any possible relationship between discrepancies and average values (Bland and Altman [2]). If the absolute value of the difference |*X*_2_ − *X*_1_| exceeds the half-width *ω*_*RB*_, those data can then be viewed as outliers from the RB.

Practitioners may choose different values of the CCC for a lower bound of excellent concordance, depending on their practical interpretation of the CCC or clinical relevancy and historical CCC values. In this paper, we employ the lower bound of the CCC of 0.75 for excellent concordance, and this threshold has been well accepted in Nickerson [12] and Rosner [13]. Assuming *C*_*b*_ = 1, the half-width of the RB is

$${\omega}_{RB}=\frac{1}{\sqrt{2}}{t}_{\nu, \alpha /2}\hat{\upsigma}.$$

Excellent concordance would not be achieved if *ρ* is lower than 0.75 since 0 < *C*_*b*_ ≤ 1. Thus, nearly 100(1 - 𝛼)% of data should be located within the RB if the CCC is at least 0.75 and *C*_*b*_ = 1. Note that random samples from a bivariate normal variable are distributed to the line

$${X}_2=\frac{\sigma_2}{\sigma_1}\ \left({X}_1-{\mu}_1\right)+{\mu}_2$$

in (*X*_1_, *X*_2_) plane, and that the slope of the best-fit line would be negative for *σ*_2_ < *σ*_1_, positive for *σ*_2_ > *σ*_1_, and 0 for *σ*_2_ = *σ*_1_ in $\left(\frac{X_1+{X}_2}{2},{X}_2-{X}_1\right)$ plane. Thus, the vertical shift of the mean difference from 0 and the slope of the best-fit line indicate the degree of heterogeneity of the two means and variances. We will investigate this in Results section.

## Results

### Simulation studies

**Fig. 2:**
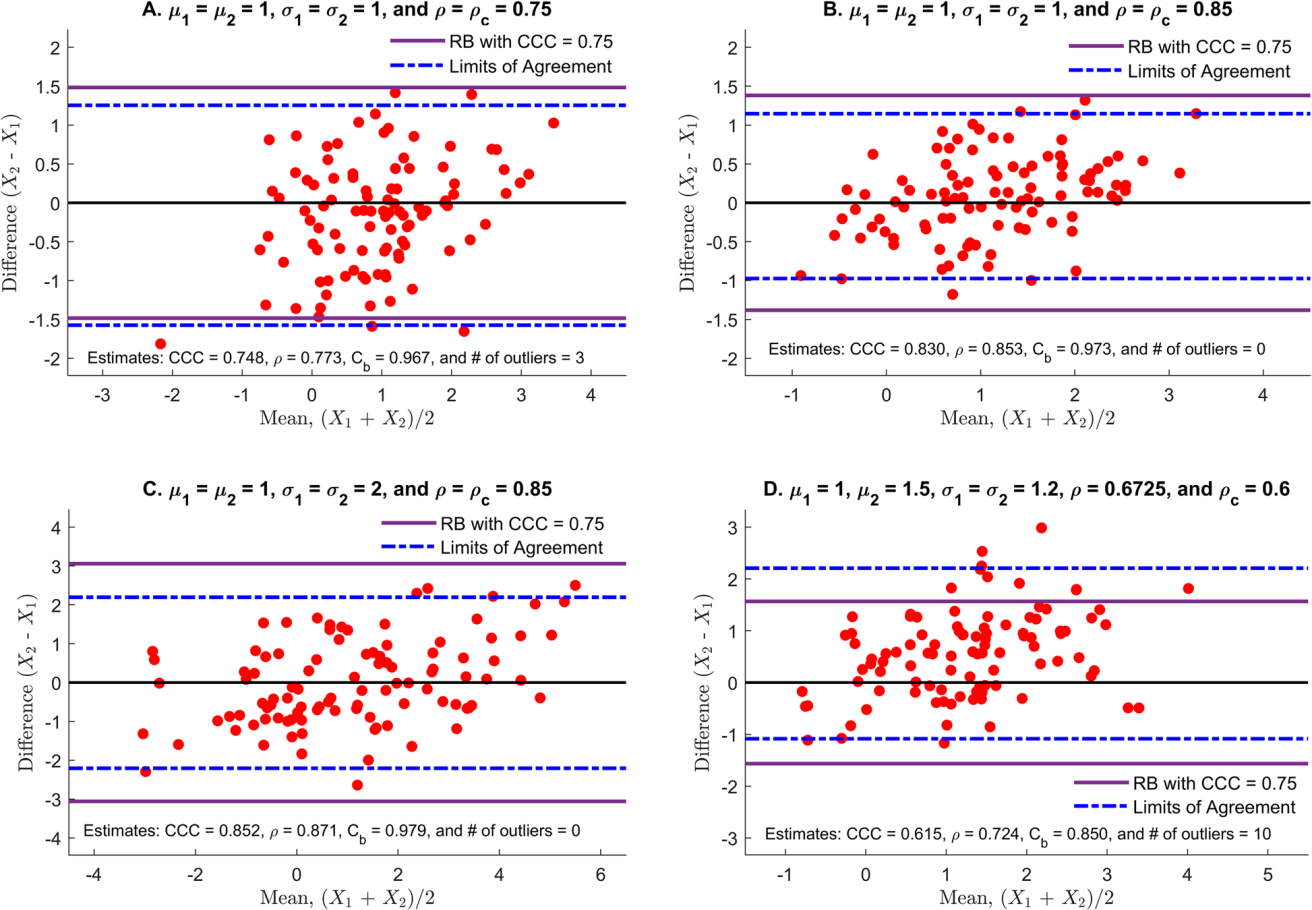
Comparisons with the limits of agreement for 4 different scenarios: scenario I (panel A, *μ*_1_ = *μ*_2_ = 1, *σ*_1_ = *σ*_2_ = 1; *C*_*b*_ = 1, and ρ = ρ_*c*_ = 0.75), scenario II (panel B, *μ*_1_ = *μ*_2_ = 1, *σ*_1_ = *σ*_2_ = 1; *C*_*b*_ = 1, and ρ = ρ_*c*_ = 0.85), scenario III (panel C, *μ*_1_ = *μ*_2_ = 1, *σ*_1_ = *σ*_2_ = 2; *C*_*b*_ = 1, and ρ = ρ_*c*_ = 0.85) and scenario IV (panel D, *μ*_1_ = 1, *μ*_2_ = 1.5, *σ*_1_ = 1, *σ*_2_ = 1.2; *C*_*b*_ = 0.8922, ρ = 0.6725; *ρ*_*c*_ = 0.6). The CCC of 0.75 is selected as a lower bound of excellent concordance. The sample size is 100

**Fig. 3:**
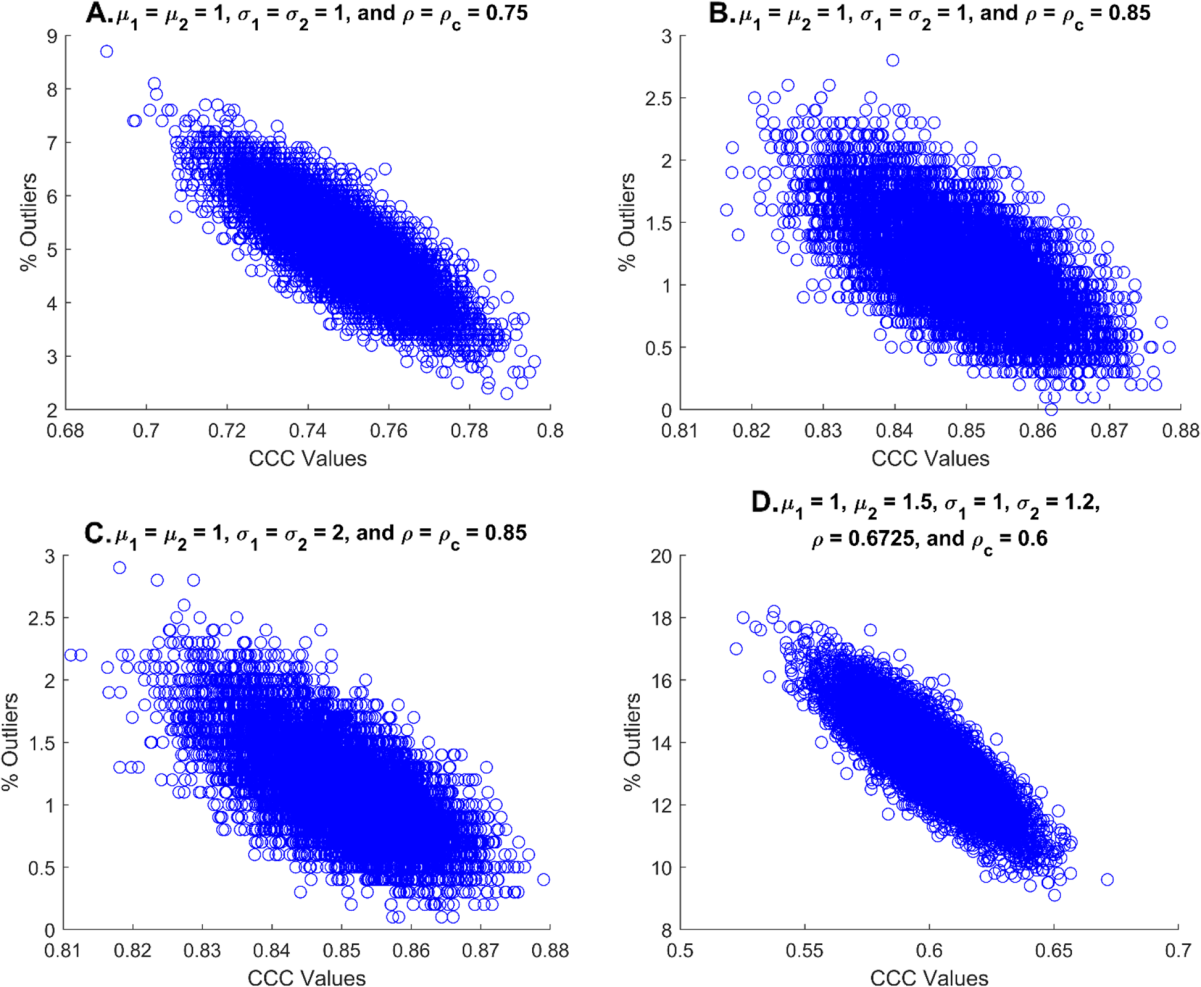
Association between the CCC values and the % of outliers for 4 different scenarios: scenario I (panel A, *μ*_1_ = *μ*_2_ = 1, *σ*_1_ = *σ*_2_ = 1; *C*_*b*_ = 1, and ρ = ρ_*c*_ = 0.75), scenario II (panel B, *μ*_1_ = *μ*_2_ = 1, *σ*_1_ = *σ*_2_ = 1; *C*_*b*_ = 1, and ρ = ρ_*c*_ = 0.85), scenario III (panel C, *μ*_1_ = *μ*_2_ = 1, *σ*_1_ = *σ*_2_ = 2; *C*_*b*_ = 1, and ρ = ρ_*c*_ = 0.85), and scenario IV (panel D, *μ*_1_ = 1, *μ*_2_ = 1.5, *σ*_1_ = 1, *σ*_2_ = 1.2; *C*_*b*_ = 0.8922, ρ = 0.6725; *ρ*_*c*_ = 0.6). The CCC of 0.75 is selected as a lower bound of excellent concordance. Under each scenario, 10,000 runs of simulation are conducted, and the sample size is 1000 to have a more accurate estimate of the % of outliers

We considered four different scenarios to illustrate the performance of our approach. Under each scenario, 10,000 runs of simulation studies were conducted to estimate the number of outliers from the RB. At each run, 1000 bivariate normally distributed random samples with 𝜌 were generated to have a more accurate estimate of the % of outliers by the method of Kim [14]. The RB in $\left(\frac{X_1+{X}_2}{2},{X}_2-{X}_1\right)$ plane is constructed by using α = 0.05 and the CCC = 0.75 as the lower limit of excellent concordance. Scenario I evaluates the number of outliers detected by the proposed method when *ρ*_*c*_ = 0.75 and *C*_*b*_ = 1. In scenarios II and III, the proposed method is compared with the LoA of the B-A plot when data are highly concordant (ρ = 0.85 and *C*_*b*_ = 1) and the common variance is either relatively small (*σ*_1_ = *σ*_2_ = 1), scenario II or large (*σ*_1_ = *σ*_2_ = 2), scenario III. The effect of heterogeneity of two variances and two means is investigated in Scenario IV. The graphical comparisons with the LoA are provided in Fig. 2, where the sample size is reduced to 100 for better visual comparisons. The association between the CCC values and the % of outliers is presented in Fig. 3.

#### Scenario I

(*μ*_1_ = *μ*_2_ = 1, *σ*_1_ = *σ*_2_ = 1; *C*_*b*_ = 1 and ρ = ρ_*c*_ = 0.75) The data are randomly distributed to the line *X*_2_ − *X*_1_ = 0 in $\left(\frac{X_1+{X}_2}{2},{X}_2-{X}_1\right)$ plane (panel A of Fig. 2). No pattern is detected and nearly 3% of data deviate from the RB, which implies that the CCC value is close to 0.75. Note that the half-width of the RB (*ω*_*RB*_= 1.48) is close to that of the LoA (ω = 1.41) since both the RB and the LoA is supposed to contain 95% of data (*S*_*d*_ = 0.713 and $\hat{\uprho}$ = 0.773). The % of outliers were strongly associated with the CCC values as shown in panel A of Fig. 3. The median CCC value was 0.75 (range: 0.69 – 0.796) while the median % of outliers was 5% (range: 2.3 – 8.7%). Particularly, there were 5335 runs with the CCC values of 0.74 to 0.76 with median % of outliers at 4.9% (range: 3.2 – 6.8%).

#### Scenario II

(*μ*_1_ = *μ*_2_ = 1, *σ*_1_ = *σ*_2_ = 1; *C*_*b*_ = 1 and ρ = ρ_*c*_ = 0.85) As in scenario I, no pattern is detected, which indicates that the bias correction factor, *C*_*b*_, would be close to 1. Compared to scenario I, no data is deviated from the RB, while approximately 95% of data is located within the LoA as depicted in panel B of Fig. 2. Based on the proposed approach, it is apparent that the agreement of the data is considerably higher than 0.75 since all data are clustered near 0 within the RB, and the slope of the best-fit line seems to be near 0. The % of outliers significantly decreases with the CCC values’ increase (Fig. 3B) and would not exceed 3% as all CCC values were greater than 0.75. Indeed, the median CCC value was 0.85 (range: 0.816 – 0.878) while the median % of outliers was 1.1% (range: 0 – 2.8%).

#### Scenario III

(*μ*_1_ = *μ*_2_ = 1, *σ*_1_ = *σ*_2_ = 2; *C*_*b*_ = 1 and ρ = ρ_*c*_ = 0.85) Compared to scenario II, the only difference is that both *σ*_1_ and *σ*_2_ are increased to 2, and the RB and the LoA are almost two-folds of the scenario II (panel C, Fig. 2). It appears less concordant than scenario II based on the half-width of the LoA, despite of the fact that the CCC of scenario III is the same as scenario II. Indeed, it appears that the degree of concordance of scenario III is the same as that of scenario II. The proposed RB method correctly reflects its concordance level with no deviates of the data points from the RB. These can be identified in panel C of Fig. 2 and panel C of Fig. 3. The median and range of the CCC values and the median and range of the % of outliers are analog to those of scenario II.

#### Scenario IV

(*μ*_1_ = 1, *μ*_2_ = 1.5, *σ*_1_ = 1, *σ*_2_ = 1.2; *C*_*b*_ = 0.8922, ρ = 0.6725; *ρ*_*c*_ = 0.6) In $\left(\frac{X_1+{X}_2}{2},{X}_2-{X}_1\right)$ plane, the data are vertically shifted (panel D, Fig. 2), and the slope of the best-fit line is slightly positive, showing *σ*_1_ < *σ*_2_. Thus, it is anticipated that the bias correction factor, *C*_*b*_, is smaller than 1. Nearly 10% of data deviates from the RB, which implies that the CCC value seems to be lower than 0.75. However, the centerline of the LoA moves up by the mean of the differences, $\overline{d}$, while about 95% of the data remains within the LoA. The half-width of the LoA (half-width = 1.646) is slightly larger than that of the proposed method (*ω*_*RB*_= 1.566). In panel D of Fig. 3, the median CCC value is 0.601 (range: 0.522 – 0.671) while the median % of outliers is 13.4% (range: 9.1 – 18.2%). Thus, the proposed method is more consistent with the CCC and provides a better visual tool for evaluating the agreement in comparison with the LoA.

In summary, nearly 95% of the data lie in the LoA for all scenarios, and the visual evaluation on agreement depends on the half-width of the LoA and the predetermined acceptable difference. If the same acceptable difference is applied to all scenarios, scenario II is the most concordant, scenario II is the most concordant, scenario I is next, and III and IV are least from the LoA approach while scenarios II and III are most concordant, scenario I is next, and IV is least based upon the proposed method. These rankings are based on % of outliers with 13.4 and 5% of the median % outliers for scenarios IV and I, respectively. We observe that the proposed method is consistent with the CCC values and is robust to the magnitude of the between-subject variability.

Graphical comparisons with the LoA approach are presented in Supplementary Fig. 1 when *X*_1_ and *X*_2_ are generated from uniform distribution and their correlation coefficients are 0.65, 0.75, 0.85, and 0.9, respectively. The sample size is 100. The random numbers are generated by the Demirtas method [15]. The % of outliers are 10, 7, 5, and 3%, when the CCC values are 0.639, 0.75, 0.853, 0.909, respectively. The half-width of the RB is not dependent on correlation *ρ* while the half-width of the LoA is inversely associated with *ρ* (the half-width of the LoA = 0.508, 0.409, 0.297, and 0.242, respectively). The association between the CCC values and the % of outliers are presented in Supplementary Fig. 2. Under each scenario, 10,000 runs of simulation (*n* = 1000 per run) were conducted: 𝜌 = 0.65 in panel A, 𝜌 = 0.75 in panel B, 𝜌 = 0.85 in panel C, and 𝜌 = 0.9 in panel D. The median CCC value and % of outliers were 0.65 (range: 0.548 – 0.732) and 11.9% (range: 8 – 16.4%) for 𝜌 = 0.65, 0.75 (range: 0.661 – 0.819) and 8.4% (range: 5.1 – 12.6%) for 𝜌 = 0.75, 0.85 (range: 0.766 – 0.914) and 5% (range: 2.4 – 8.9%) for 𝜌 = 0.85, and 0.9 (range: 0.839 – 0.947) and 3.3% (range: 1.1 – 5.7%) for 𝜌 = 0.9, respectively. Thus, slightly more outliers are observed than those of bivariate normal data if data are uniformly distributed.

### Applications to real data

A peak expiratory flow rate (PEFR) study data in Bland and Altman’s paper [1] and the Radiomics features extracted from 3D CT images in Balagurunathan et al. [7] are investigated as real examples below.

#### Example 1 (PEFR data)

The PEFR was measured using two different types of equipment: a large Wright peak flow meter and a mini Wright peak flow meter. There were two measurements for each meter, as shown in Supplementary Table 1. Only the first measurement by each meter is used for the comparison of our proposed method with the LoA, which is obtained as.

$\overline{d}\pm {t}_{16,0.025}{S}_d=-2.12\pm 82.18$ (*l*/ *min* ).The boundary lines of the RB are.

**Fig. 4:**
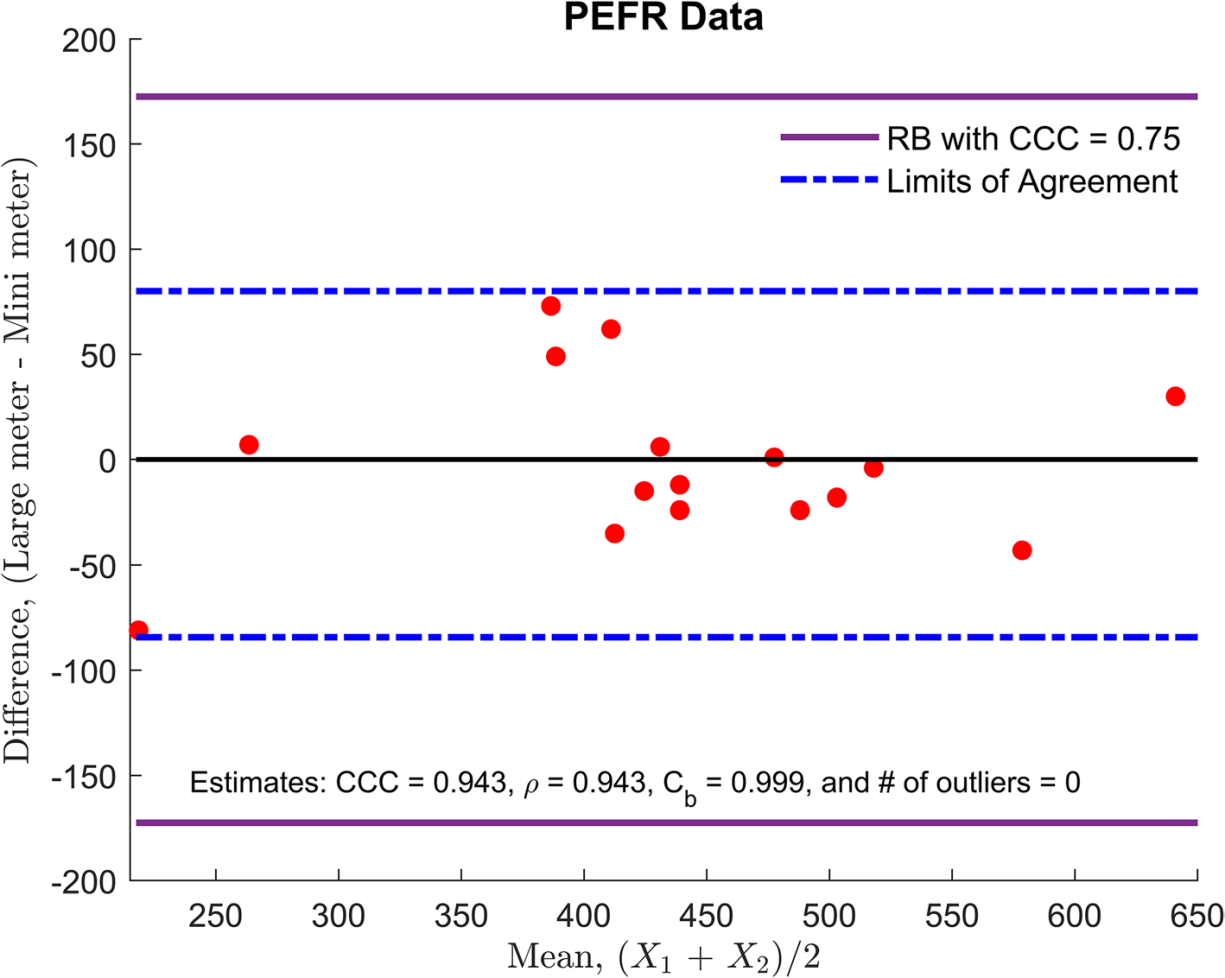
Peak Expiratory Flow Rate (PEFR) data analysis: the comparison of the reference band (RB) with the limits of agreement. A lower bound of excellent concordance is set up at 0.75

${\omega}_{RB}=\pm \frac{1}{\sqrt{2}}\ {t}_{\mathrm{16,0.025}}\hat{\upsigma}=\pm 172.53$ (*l*/ *min* ),in $\left(\frac{X_1+{X}_2}{2},{X}_2-{X}_1\right)$ plane. As depicted in Fig. 4, the half-width of the LoA is approximately two-folds of the RB. All data are clustered in the RB, implying that the CCC value would be considerably greater than 0.75 and that the two meters have an excellent concordance from the scaled index perspective. Note that estimates of the CCC, the Pearson correlation coefficient, and the bias correction factor are 0.943, 0.943, and 0.999, respectively, due to the large between-subject variability. However, the mini meter is unacceptable for clinical purposes because the half-width of the LoA (±82.18) is too wide to be considered as evidence of the lack of reproducibility.

#### Example 2 (Radiomics data)

In Balagurunathan et al. study [7], authors developed and identified a set of features extracted from CT images that can be converted into quantifiable and minable data as a potential prognostic and predictive biomarker of clinical outcomes. The unenhanced thoracic CT images for 32 patients in test-retest settings were acquired within 15 min of each other, using the same CT scanner. All patients had a primary pulmonary tumor of 1 cm or larger. A total of 64 lesions (2 per patient) were segmented, and a total of 219 3D features were extracted from CT scans. Two segmentation methods, manual and automatic single-click ensemble segmentation developed by Balagurunathan et al., were used to get the correct segmentation boundaries of tumors. These 219 features can be broadly divided into two classes: non-texture and texture features. Non-texture features include tumor size, shape, and location description, while texture features include pixel histogram, run length, co-occurrence, Laws, and wavelet-based features (see details in Balagurunathan et al. [7]). The first step of the process is to screen out less reproducible features. Unlike the PEFR study, it is impractical to determine the acceptable difference for assessing the agreement between two observations. Thus, the scaled index such as CCC would be a reasonable measure for assessing agreement.

**Fig. 5:**
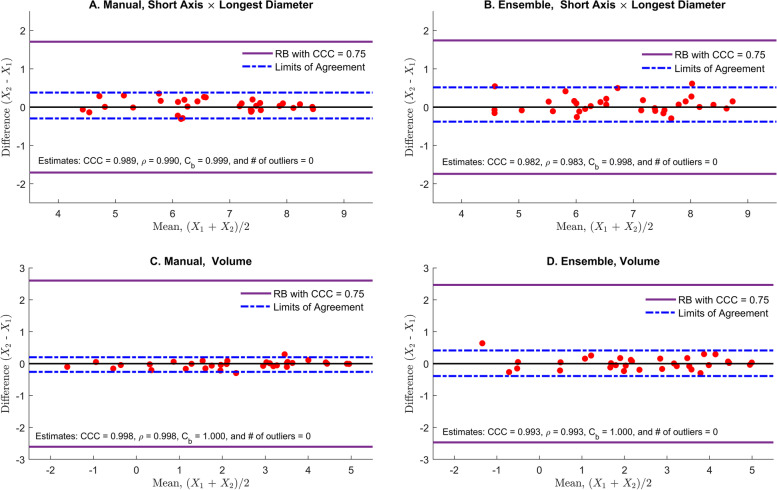
Radiomics data analysis: the comparison of the reference band (RB) with the limits of agreement (LoA). Shortest × longest diameter from manual segmentation (panel A), shortest × longest diameter from ensemble segmentation (panel B), volume from manual segmentation (panel C), and volume from ensemble segmentation (panel D). All data are log-transformed

This paper considers two non-texture features, shortest × longest diameter and volume, out of 219 features for each segmentation method (manual and ensemble segmentation). The log-transformation is taken to improve the normality. The estimated CCC values, ${\hat{\rho}}_c$, of two features obtained by two segmentation methods are very close to 1 (Table 1), and the graphical evaluation of agreement is presented in Fig. 5. The CCC value of 0.75 is selected as the lower limit of excellent concordance. As shown in Fig. 5, all data are clustered near 0 within the RB, all CCC values are considerably larger than 0.75, and it is anticipated from the visual evaluation that the agreement of volume by manual segmentation (panel C) is the highest while shortest × longest diameter by ensemble segmentation (panel B) is the lowest among them, which is consistent with the CCC values, ${\hat{\rho}}_c$ (Table 1).

**Table 1:** Radiomics Data Analysis; agreement of the features obtained from manual and ensemble segmentation. Data are all log-transformed

Features	Manual			Ensemble		
	${\hat{\rho}}_c$	$\hat{\rho}$	${\hat{C}}_b$	${\hat{\rho}}_c$	$\hat{\rho}$	${\hat{C}}_b$
Short Axis × Longest Diameter [mm^2^]	0.9895	0.9902	0.9992	0.9818	0.9835	0.9983
Volume [cm^3^]	0.9977	0.9981	0.9997	0.9933	0.9934	0.9999

## Discussion and conclusions

The Bland-Altman (B-A) plot with the limits of agreement (LoA) has been widely used as not only an unscaled agreement index but also as a visual tool for assessing agreement. The agreement is evaluated by comparing the acceptable difference with the LoA, an unscaled index. If an acceptable difference cannot be determined or the difference between measurements may not be interpretable, the scaled indices such as CCC or ICC may be used to assess the agreement. Despite its popularity, the LoA in the B-A plot may not be associated with the scaled indices, particularly when the common variance is large but two measurements are highly concordant (scenario III vs scenario II). To our knowledge, there is no visual tool available in practice that is associated with the CCC value. This paper proposes a novel, CCC-based reference band (RB) as a visual tool for assessing agreement. The simulation studies show that our visual tool is consistent with the CCC value. If data are uniformly distributed, slightly more outliers of the RB are detected than those of bivariate normal data. Note that the RB is derived from the assumption that *σ* = *σ*_1_ = *σ*_2_. If this assumption does not hold, the width of the RB may not be reliable, and the number of outliers may not be consistent with the CCC value. Thus, the test for the homogeneity of two variances is recommended prior to applying this method in practice. However, the test for the homogeneity of two means is not necessary since the difference of two means does not affect the validity of the half-width of the RB. We also hope that the proposed method can provide practitioners with additional useful information such as recognition of patterns and identification of outliers in data.

## Supplementary Information

**Additional file 1: Supplementary Table 1.** Peak Expiratory Flow Rate (PEFR; *l*/*min*) measured with Wright peak flow and Mini Wright flow meters. **Supplementary Figure 1.** Comparisons with the limits of agreement for 4 different scenarios. **Supplementary Figure 2.** Association between the CCC values and the % of outliers for 4 different scenarios.

## Data Availability

PEFR Data are presented in Supplementary Table 1. *MATLAB* programs and real data files used for this paper are available at GitHub (https://github.com/JPKim89/Graphical-Evaluation.git) or upon request at Jongphil.Kim@moffitt.org. Radiomic data may be available upon Dr. Robert Gillies’ approval.

## Abbreviations

B-A: Bland-Altman
CCC: Concordance correlation coefficient
CP: Coverage probability
ICC: Intraclass correlation coefficient
LoA: Limits of agreement
PEFR: Peak expiratory flow rate
RB: Reference band
TDI: Total deviation index
