# Supplementary material for: A novel graphical evaluation of agreement
Source: BMC Med Res Methodol. 2022 Feb 20;22:51. doi: 10.1186/s12874-022-01532-w (PMC8858529; doi:10.1186/s12874-022-01532-w)
Supplement: Supplementary file 1 — Additional file 1: Supplementary Table 1. Peak Expiratory Flow Rate (PEFR; l/min) measured with Wright peak flow and Mini Wright flow meters. Supplementary Figure 1. Comparisons with the limits of agreement for 4 different scenarios. Supplementary Figure 2. Association between the CCC values and the % of outliers for 4 different scenarios. [file 12874_2022_1532_MOESM1_ESM.docx]

Supplementary Table 1. Peak Expiratory Flow Rate (PEFR; $l/min$) measured with Wright peak flow and Mini Wright flow meters

| Subject | Large Wright Peak Flow Meter | | Mini Wright Peak Flow Meter | | Large meter - Mini meter |
| --- | --- | --- | --- | --- | --- |
|  | 1st PEFR | 2nd PEFR | 1st PEFR | 2nd PEFR |  |
| 1 | 494 | 490 | 512 | 525 | -18 |
| 2 | 395 | 397 | 430 | 415 | -35 |
| 3 | 516 | 512 | 520 | 508 | -4 |
| 4 | 434 | 401 | 428 | 444 | 6 |
| 5 | 476 | 470 | 500 | 500 | -24 |
| 6 | 557 | 611 | 600 | 625 | -43 |
| 7 | 413 | 415 | 364 | 460 | 49 |
| 8 | 442 | 431 | 380 | 390 | 62 |
| 9 | 650 | 638 | 658 | 642 | -8 |
| 10 | 433 | 429 | 445 | 432 | -12 |
| 11 | 417 | 420 | 432 | 420 | -15 |
| 12 | 656 | 633 | 626 | 605 | 30 |
| 13 | 267 | 275 | 260 | 227 | 7 |
| 14 | 478 | 492 | 477 | 467 | 1 |
| 15 | 178 | 165 | 259 | 268 | -81 |
| 16 | 423 | 372 | 350 | 370 | 73 |
| 17 | 427 | 421 | 451 | 443 | -24 |
| Mean | 450.35 | 445.41 | 452.47 | 455.35 | -2.12 |
| SD | 116.31 | 119.61 | 113.12 | 111.32 | 38.77 |


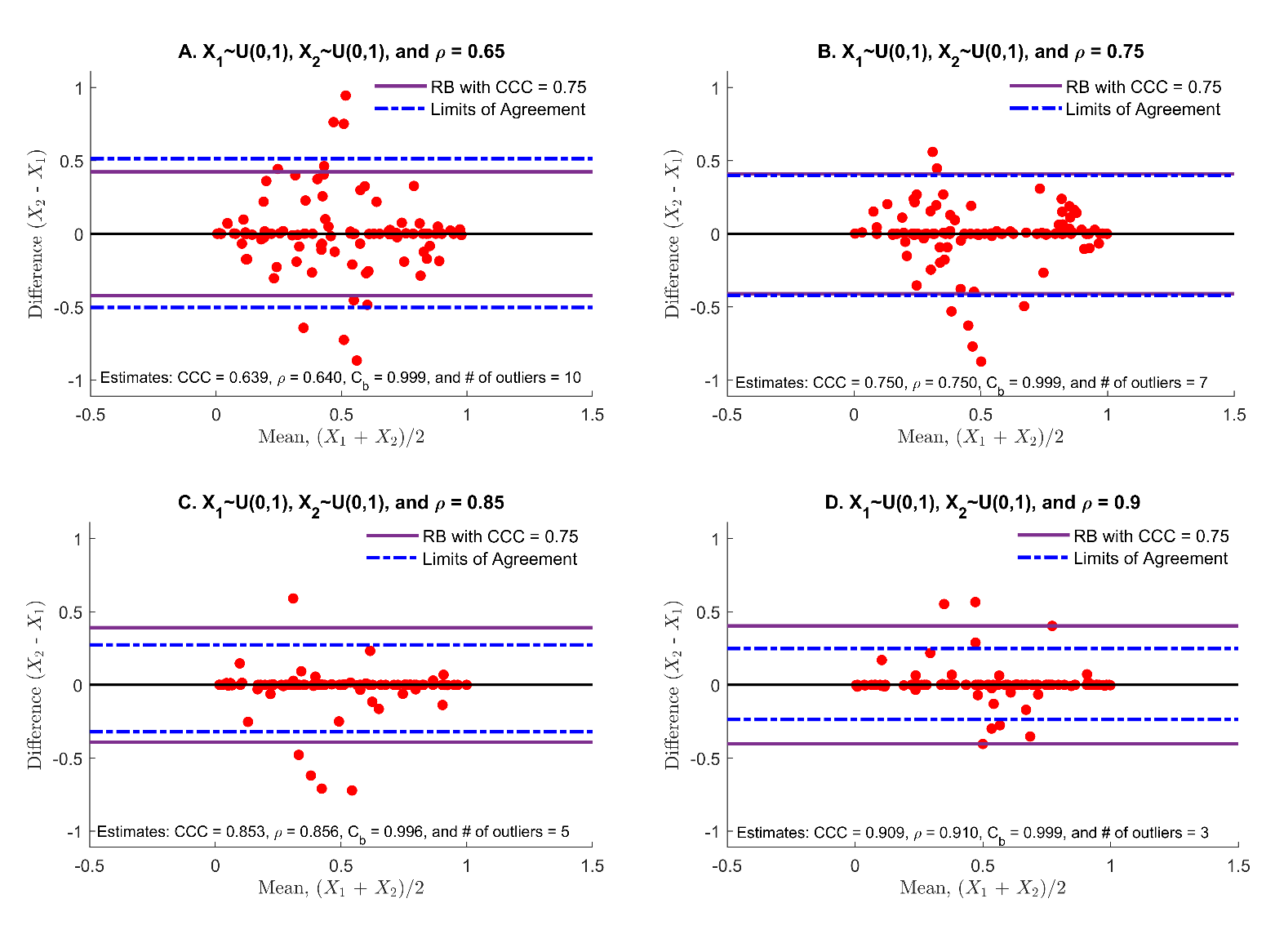


Supplementary Figure 1. Comparisons with the limits of agreement for 4 different scenarios: panel A ($C_{b}=1$, $\rho=0.65$), panel B ($C_{b}=1$, $\rho=0.75$), panel C ($C_{b}=1$, $\rho=0.85$), and panel D ($C_{b}=1,$ $\rho=0.9$). The CCC of 0.75 is selected as a lower bound of excellent concordance. The sample size is 100. $X_{1}$ and $X_{2}$ are generated from uniform distribution by the Demirtas method. The % of outliers are 10%, 7%, 5%, and 3%, respectively. The half-width of the RB is not dependent on the correlation $\rho$ while the half-width of the LoA is inversely associated with $\rho$ (the half-widths of the LoA are 0.508, 0.409, 0.297, and 0.242, respectively).


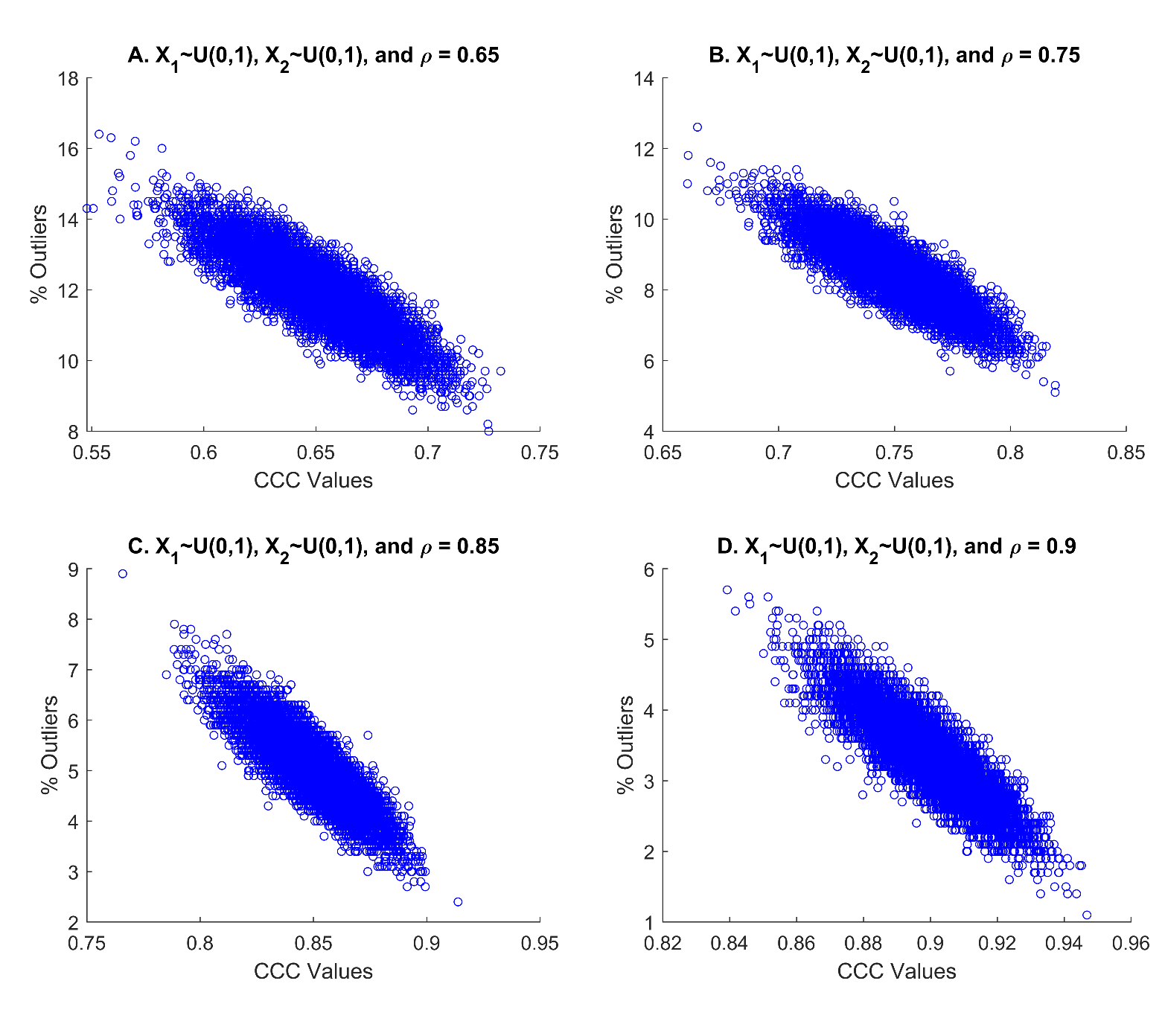


Supplementary Figure 2. Association between the CCC values and the % of outliers for 4 different scenarios: panel A ($C_{b}=1$, $\rho=0.65$), panel B ($C_{b}=1$, $\rho=0.75$), panel C ($C_{b}=1$, $\rho=0.85$), and panel D ($C_{b}=1,$ $\rho=0.9$). The CCC of 0.75 is selected as a lower bound of excellent concordance. Under each scenario, 10,000 runs of simulation are conducted, and the sample size of each run is 1,000. $X_{1}$ and $X_{2}$ are generated from uniform distribution by the Demirtas method.
